# Assessment of Clinical Predictability of Overbite Reduction and Curve of Spee Levelling in Clear Aligner Treatment

**DOI:** 10.1111/ocr.12894

**Published:** 2025-01-08

**Authors:** Simona Dianiskova, Roberto Rongo, Domenico Sirignano, Rosalinda D'Amelio, Giorgio Oliva, Rosa Valletta, Vincenzo D'Antò

**Affiliations:** ^1^ Medical Faculty, Department of Orthodontics Slovak Medical University Bratislava Slovakia; ^2^ Department of Neuroscience, Reproductive Sciences and Oral Sciences, Section of Orthodontics University of Naples Federico II Naples Italy

## Abstract

**Objective:**

This retrospective study aims to assess the predictability of Overbite (OVB) reduction and Curve of Spee (COS) levelling in patients with deep bite malocclusion treated with Clear Aligner Therapy (CAT). The research evaluates the accuracy of the prescribed movements in growing and non‐growing patients.

**Materials and Methods:**

Thirty‐six patients treated with Invisalign from January 2018 using SmartTrack were included. Digital models at pre‐treatment, virtual plan and post‐treatment phases were collected and analysed using Geomagic Control X. Accuracy was assessed for COS levelling, OVB reduction and individual tooth movements. Statistical analyses included descriptive statistics, paired *t*‐tests and linear regression between accuracy of the movements and the age of the patient.

**Results:**

The study found that the prescribed movements in ClinCheck often overestimated the achieved outcomes. The mean accuracy for COS levelling was 62%, and for OVB reduction, it was 61%. No significant association was observed between the accuracy and the age of the patient.

**Conclusion:**

Clear Aligner Therapy demonstrated moderate accuracy in achieving prescribed movements for COS levelling and OVB reduction. Clinicians should exercise caution in virtual treatment planning and may need to consider potential modifications to software projections to enhance desired outcomes. Further research comparing different aligner protocols and brands is warranted to advance understanding and improve treatment predictability.

## 1 Introduction

Deep bite is a malocclusion characterised by an increased overbite (OVB) [1] and often also by an exaggerated Curve of Spee (COS), which must be levelled to achieve a good occlusion [2]. A skeletal, dental, or dentoskeletal problem can cause a deep bite, and treatment options include the extrusion of posterior teeth, intrusion of lower and upper incisors, distal tipping of posterior teeth and proclination of anterior teeth. Most of the time it is treated with a combination of these movements, depending on the age, the diagnosis and the aesthetic of the patient [3].

Clear aligner therapy (CAT) encompasses various appliances with different mechanisms of action and construction methods [4]. Despite the many advantages of CAT [5] and the innovations in materials and auxiliaries, it still faces challenges in controlling certain tooth movements [6]. For example, elastics might be necessary when there is also a Class II malocclusion [7, 8], and precision bite ramps are the elective choice to create proper space for lateral sector extrusion [9, 10].

Over the years, a substantial body of research has focused on the effectiveness of teeth movement with CAT [6, 11, 12, 13, 14, 15, 16, 17, 18, 19, 20]. Kravitz et al. updated the previous studies where they compared programmed tooth movements on ClinCheck to actual tooth movements and found that overall, the mean accuracy with Invisalign rose only to 50% [14] from the previous 41% [11]. According to Rossini et al., the most difficult movement to control is extrusion (30% accuracy), while intrusion is easier to achieve, with the highest accuracy for maxillary (45%) and mandibular (47%) central incisors [21]. Usually COS and OVB reduction require both movements; therefore, the accuracy of this correction is under discussion [22, 23].

Indeed, part of the scientific research has focused solely on the accuracy of clear aligners in treating deep bites [22, 23, 24, 25, 26, 27, 28, 29, 30]. Blundell et al. assessed that ClinCheck overestimated OVB correction; the Invisalign appliance expressed 39.2% of the planned overbite reduction, compared to the result prescribed with the ClinCheck software. Their study highlights that the deeper the OVB pre‐treatment is, the more difficult it can be to achieve OVB reduction [23]. Regarding COS, ClinCheck overestimated mandibular COS levelling in 86% of patients, with a prescription accuracy of 35% [22]. All these authors agreed on the need for overcorrections and additional refinements in most patients with deep bites, even with the newest enhancements [31].

However, there is still a lack of information on the accuracy of CAT in COS levelling and OVB reduction. Hence, the present study wants to evaluate how well CAT works with these movements when employed to treat deep bite. The aim is to check if what is planned with ClinCheck is obtained, or if overcorrections are actually nearly always necessary.

## 2 Materials and Methods

The Ethics Committee of the University of Naples Federico II (Italy) approved the research protocols with the following codes: 179 2023 /181 2023.

The sample size calculation was based on existing studies in the literature, which focused on the difference between planned and achieved OVB. In order to attain a statistical power of 90%, a paired two‐tailed *t*‐test with an acceptable margin of difference of 0.5 mm was employed, the sample size should consist of 20 patients [23].

Thirty‐six patients (19 males and 17 females; mean age: 23.6 ± 10.8 years, range 11–45 years) were selected according to the following inclusion criteria:

- treated with Invisalign from January 2018 using SmartTrack;
- dual arch treatment;
- completed initial treatment with a minimum of 14 aligners (42.96 ± 12.41 aligners);
- availability of pre‐treatment and progress digital scans;
- all mandibular teeth erupted (except for third molars);
- overbite > 4 mm;
- non‐extractive orthodontic treatment;
- OVB reduction and COS levelling prescribed in the ClinCheck treatment plan.

The exclusion criteria were as follows:

- uncooperative patients (patients who did not attend each appointment regularly or who showed misfitting aligners);
- deep bite > 8 mm;
- extractive orthodontic treatment;
- patients requiring orthognathic surgery;
- incomplete registration of the distobuccal cusp of the second molars;
- any syndrome, craniofacial disorder or medical condition affecting bone metabolism or tooth movement.

All patients used clear aligners for 22 h daily, changed them every 10 days, and no particular staging protocol was prescribed for anterior intrusion. Incisor bite ramps were used if a 2 mm anterior intrusion (or more) was prescribed, and Class II elastics were prescribed if class correction was an objective of the treatment. All patients were instructed to check the fit of their aligners prior to changing them and to contact the clinic if misfitting occurred. If significant misfitting was found during appointments, patients were classified as uncooperative, as mentioned in the exclusion criteria. The protocol did not include overcorrections of anterior OVB (no cases planned with inverted OVB); all cases had a planned OVB between 1 and 3 mm.

For COS levelling, the protocol did not include any premolar extrusion over 1 mm as a form of overcorrection: these data refer to the ClinCheck movement table and differ from those of this study, as the latter are affected by the change in the OP.

Fourteen patients used class II elastics applied directly to a button positioned on the molar and 19 patients used bite ramps.

Three digital dental models (STL files) of the maxillary and mandibular arches were collected for each patient: pre‐treatment (T0), the virtual treatment plan (T1) and the post‐treatment digital model at the end of the COS levelling and OVB reduction phase (T2). The pre‐treatment and post‐treatment digital models were acquired using an intraoral scanner (iTero Element scanner; Align Technologies, San Jose, Calif, USA). The STL file of the virtual plan was exported to examine the accuracy of the planned movements.

The digital dental models were imported into Geomagic Control X (version 2022.1.0.70, 3D Systems, Rock Hill, SC, USA). All teeth were segmented in T0. The segmentation enabled the performance of a partial surface‐based best fit with 50 iterations for each tooth to identify the same landmarks on the STL files [32].

**FIGURE 1:**
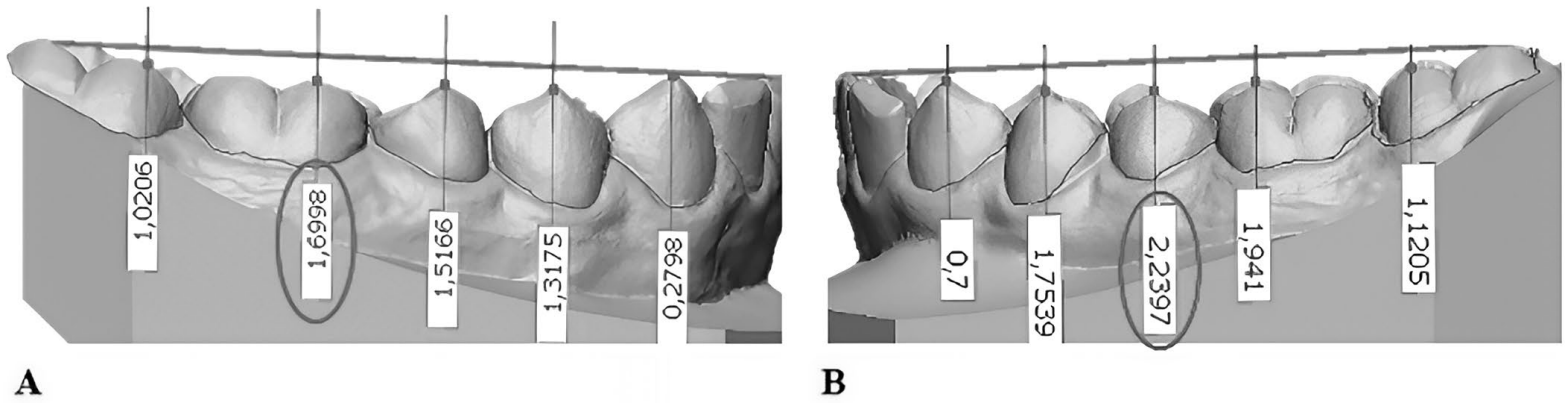
Measurement of the depth of the curve of Spee, identified by averaging the deepest vertical distance to the OP on the right (A) and on the left side (B).

To determine COS depth, the tip of the buccal cusps of second molars, the mesiobuccal cusp of first molars, the buccal cusps of premolars, the cusp of canines and the midpoint between the edges of the two mandibular central incisors were identified. It was then necessary to establish an Occlusal Plane (OP), defined by the distobuccal cusps of both second molars and the midpoint between the edges of the two mandibular central incisors [33]. The tool ‘digital calliper’ of Geomagic Control X was used to measure vertical distances of the cusps previously selected (except for the distobuccal cusp of second molars) to the OP. The COS value was identified for each single model by averaging the deepest value on both the right and left sides (Figure 1) [22].

**FIGURE 2:**
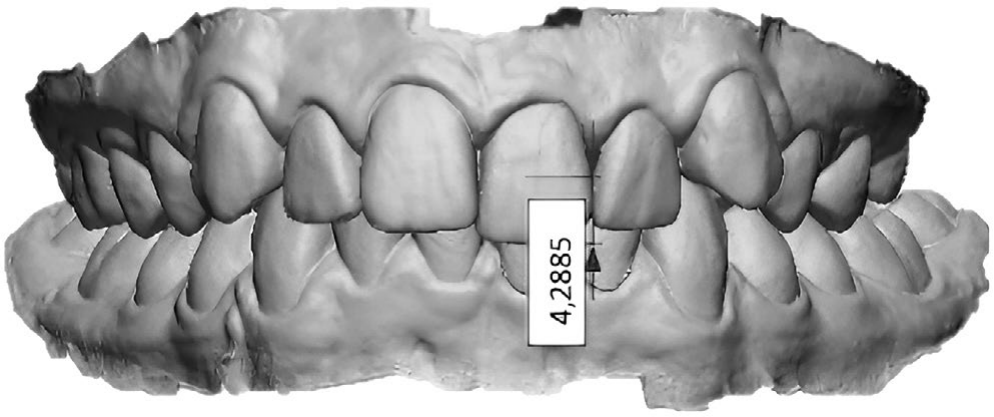
Vertical linear distance between the vector and the midpoint of the incisal edge of the ipsilateral maxillary incisor used to measure OVB depth.

Whereas, regarding the OVB measurement, STL files of the upper and lower arches were put into occlusion and imported into Geomagic Control X. Here, after segmenting all the teeth in T0, the midpoints of the incisal edges of the mandibular central and lateral incisors and the midpoint of the incisal edge of the ipsilateral maxillary central incisor were identified. OVB was measured on the side with the highest value. A vector passing through the midpoints of the incisal edges of the two mandibular incisors was constructed, and the vertical linear distance between this vector and the midpoint of the incisal edge of the ipsilateral maxillary incisor was used to measure OVB depth (Figure 2) [23].

The same measurements were made on T1 and T2 for both COS and OVB evaluation.

T0 and T1 were compared to establish the amount of Prescription, and T0 and T2 to determine the Achieved Movement.

COS levelling and OVB reduction, as well as the levelling of each tooth, were analysed according to the following variables:

- Prescription was the distance measured when comparing pre‐treatment (T0) and virtual plan digital models (T1).
- Achieved Movement was the distance measured when comparing pre‐treatment (T0) and post‐treatment digital models (T2).
- Accuracy, expressed as a percentage of achieved versus planned, was calculated as follows:

Accuracy = (Achieved Movement/Prescription) × 100.

The overall accuracy was calculated as the mean of the accuracy for each outcome.

The normality of the sample's distribution of cases was assessed using the Shapiro–Wilk test.

The study employed the ICC statistical method to assess the reproducibility of measurements. Four weeks after the first evaluation, 40% of the digital dental models were re‐analysed by the same operator and again by a different operator with experience in Geomagic Control X. Statistical analysis was performed using the SPSS software package (IBM, Chicago, IL, USA).

Statistics were computed for Prescription, Achieved Movement and accuracy. A paired two‐tailed *t*‐test was employed to evaluate the statistical significance of differences between Prescription and Achieved Movement. The significance level was set at 0.05, and the Benjamini–Hochberg method was used to adjust the *p*‐value.

A series of linear regressions were performed to assess the influence of the age of the patients (taken as independent variable) on the accuracy (dependent variable) of each movement. The significance level was set at 0.05.

## 3 Results

Table 1 provides the ICC values for intra‐examiner and inter‐examiner evaluations. An ICC value below 0.500 indicates poor agreement, while a value above 0.900 indicates excellent agreement. All the ICC values are above the excellent agreement level.

**TABLE 1:** Intraexaminer and interexaminer reliability.

Intraexaminer				Interexaminer		
	Pre‐treatment	ClinCheck	Post‐treatment	Pre‐treatment	Clincheck	Post‐treatment
COS	0.988	0.966	0.973	0.994	0.978	0.960
Canine	1.000	0.997	0.996	0.997	0.991	0.990
1 PM	0.992	0.968	0.989	0.991	0.998	0.993
2 PM	0.997	0.996	0.990	0.999	0.995	0.998
1 M	0.997	0.991	0.993	0.997	0.995	0.990
2 M	0.997	0.982	0.997	0.954	0.995	0.991
OVB	0.983	0.998	0.972	0.956	0.989	0.959

Abbreviations: 1 M, mandibular first molar; 1 PM, mandibular first premolar; 2 M, mandibular second molar; 2 PM, mandibular second premolar.

The average outcome measurements for pre‐treatment, post‐treatment and the planned outcome (ClinCheck) are reported in Table 2. The average overbite (OVB) was 5.03 mm, while the planned outcome was 1.80 mm. However, the treatment was able to achieve an overbite of 3.03 mm. The pre‐treatment average depth of the curve of Spee (COS) was 2.48 mm. After the treatment, the average COS depth was 1.61 mm, compared to the expected 1.18 mm. The first molar had the greatest distance from the OP. Table 3 presents the means for both Prescription and Achieved Movement and the resulting accuracy. Statistical analysis revealed a significant difference between Prescription and Achieved Movement in all subgroups. COS requires an average levelling of 1.30 mm but achieves only 0.87 mm of movement. The premolars and first molars were the most levelled teeth to the occlusal plane (OP). Regarding OVB, an OVB opening of 3.23 mm was prescribed, while the Achieved Movement is 2.00 mm.

**TABLE 2:** Descriptive statistics of outcomes.

	Pre‐treatment						Clincheck						Post‐treatment					
	Min	Max	Mean	SE. Mean	std. Dev	SW test	Min	Max	Mean	SE. Mean	std. Dev	SW test	Min	Max	Mean	SE. Mean	std. Dev	SW test
COS	0.42	5.23	2.48	0.15	0.88	0.91	−1.32	2.16	1.18	0.11	0.66	0.89	−0.27	2.99	1.61	0.12	0.71	0.97
Canine	−1.50	2.88	0.30	0.15	0.90	0.95	−1.22	0.13	−0.40	0.06	0.34	0.97	−1.20	0.87	−0.17	0.08	0.47	0.99
1 PM	−0.59	2.89	1.45	0.13	0.79	0.98	−1.77	0.97	−0.28	0.09	0.53	0.97	−1.31	1.97	0.24	0.12	0.71	0.99
2 PM	−0.22	5.23	1.98	0.16	0.95	0.94	−1.70	1.76	0.40	0.11	0.68	0.96	−1.12	2.99	0.85	0.13	0.76	0.96
1 M	0.42	3.74	2.32	0.11	0.69	0.98	−1.32	2.22	1.17	0.12	0.69	0.92	−0.27	4.25	1.60	0.14	0.84	0.95
2 M	−0.28	2.55	0.97	0.08	0.51	0.86	−0.98	1.59	0.50	0.09	0.51	0.97	−0.65	2.36	0.67	0.09	0.53	0.93
OVB	4.15	6.69	5.03	0.12	0.70	0.92	0.09	3.50	1.80	0.13	0.78	0.99	1.12	6.08	3.03	0.18	1.08	0.95

*Note*: All measurements are in millimetres. For COS and individual teeth, a positive value signifies a position below the occlusal plane, while a negative value indicates a position above it.

Abbreviations: 1 M, mandibular first molar; 1 PM, mandibular first premolar; 2 M, mandibular second molar; 2 PM mandibular second premolar; SE, Mean, Standard Error of the Mean; std. Dev, standard deviation; SW test, Shapiro–Wilk test.

**TABLE 3:** Descriptive statistics of Prescription and Achieved Movement.

	Prescription						Achieved movement						AM vs. *p*		Accuracy
	Min	Max	Mean	SE. Mean	std. Dev	SW test	Min	Max	Mean	SE. Mean	std. Dev	SW test	*p*	P.adj	Mean
COS	−3.54	0.24	−1.30	0.14	0.84	0.94	−2.76	0.32	−0.87	0.13	0.81	0.93	< 0.000	< 0.000	62%
Canine	−3.35	0.91	−0.70	0.14	0.82	0.95	−3.44	0.74	−0.48	0.14	0.82	0.92	< 0.000	< 0.000	60%
1 PM	−3.64	−0.27	−1.73	0.13	0.75	0.98	−3.47	0.53	−1.21	0.15	0.89	0.99	< 0.000	< 0.000	62%
2 PM	−3.46	0.24	−1.58	0.14	0.86	0.97	−3.05	0.90	−1.14	0.14	0.87	0.99	< 0.000	< 0.000	57%
1 M	−2.34	0.24	−1.15	0.11	0.65	0.98	−2.33	0.51	−0.72	0.12	0.70	0.97	< 0.000	< 0.000	58%
2 M	−1.19	−0.03	−0.47	0.05	0.29	0.95	−0.77	−0.02	−0.31	0.03	0.20	0.96	< 0.000	< 0.000	83%
OVB	−5.31	−1.19	−3.23	0.15	0.89	0.99	−4.90	−0.05	−2.00	0.18	1.08	0.96	< 0.000	< 0.000	61%

*Note*: AM versus *p*, Achieved Movement versus Prescription; P.adj, adjust *p*‐value according to the Benjamini–Hochberg method. All measurements are in millimetres. A positive change indicates an intrusive movement relative to the OP. A negative change indicates an extrusive movement relative to the OP.

Abbreviations: 1 M, mandibular first molar; 1 PM, mandibular first premolar; 2 M, mandibular second molar; 2 PM mandibular second premolar; SE. Mean, Standard Error of the Mean; std. Dev, standard deviation; SW test, Shapiro–Wilk test.

**FIGURE 3:**
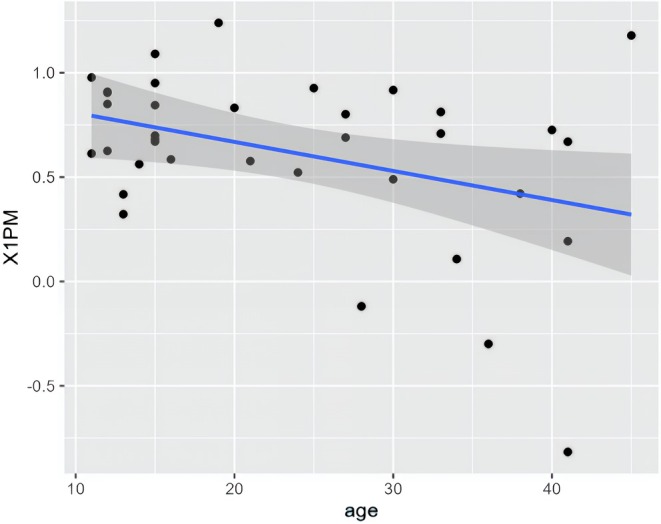
Scatter Plot of the linear regressions between age and accuracy of first premolar.

The highest accuracy appears to be associated with the second molar at 83%, followed by the first premolar at 62%, while the second premolar exhibits the lowest accuracy at 57%. For COS and OVB correction, the accuracy was 62% and 61%, respectively. As regard the accuracy level and age, only the 1 PM showed statistically significant association: as the age increases, the accuracy decreases, as shown in Figure 3.

## 4 Discussion

The objective of this study is to evaluate the effectiveness of OVB correction and COS levelling with orthodontic aligners. To the best of our knowledge, this is the first manuscript dealing with the accuracy of both overbite reduction and curve of Spee levelling in patients of different ages. In most cases (89%, or 32 out of 36, for COS; 94%, or 34 out of 36, for OVB), the prescriptions overestimated the extent of bite opening and COS levelling obtained at the end of these phases. Indeed, on average, in these patients, the accuracy of COS levelling was 62% (95% CI, 42%–83%).

The study by Goh et al. [22] analysed initial scans, ClinCheck predictions and post‐treatment scans and found an accuracy of 35%. Concerning the relative extrusion of posterior teeth in relation to the OP, Goh et al. observed that the lowest accuracy was detected in the first molar at 31%, while the second molar showed the highest accuracy at 52%. In our study, the second molar exhibited the highest accuracy with 83% (95% CI, 64%–102%); the first molar demonstrated superior accuracy with 58% (95% CI, 35%–81%). However, the second premolar had the lowest accuracy at 57% (95% CI, −29% to 84%). Premolars had the highest Prescription for relative extrusion to the occlusal plane in both studies. In this study, the prescribed extrusion for the first premolar was 1.73 mm (95% CI, 1.47–1.98 mm), and for the second premolar, it was 1.57 mm (95% CI, 1.29–1.86 mm).

When analysing the results, it should be considered that the second molar is used to construct the reference plane; greater accuracy can be attributed to this. The substantial discrepancy observed compared to that reported by the authors may indeed be attributed to the broad inclusion criteria adopted in this study. The intentional decision not to narrow down the inclusion criteria, as underscored by Goh et al., was made to avoid excluding cases treated with auxiliaries that are routinely used in CAT. The effects of auxiliaries can be of various types. For instance, inter‐arch elastics applied directly to a button positioned on the molar can facilitate extrusion; bite ramps, as indicated by Blundell et al. [26, 27] do not enhance incisor intrusion; however, they might be more effective in levelling the COS by mitigating masticatory forces during occlusion.

In the study by Goh et al. [22], the mean levelling of the COS using ClinCheck was prescribed to 42% of the initial depth, while the actual outcome only achieved 15% levelling of the initial depth. Similarly, in this study, the prescribed levelling of the COS using ClinCheck accounted for 52% of the initial depth, while the actual achieved outcome represented only 35% of the initial levelling. These findings highlight a significant discrepancy between the prescribed levelling of the COS by ClinCheck and the actual expression achieved using the Invisalign appliance.

Castroflorio and colleagues [34] analysed the accuracy of the planned movements in different directions. Although percentage variations were not reported, the analysis of millimetric variations reveals a greater occlusal load on the first molar, reducing the predictability of planned extrusion.

Regarding the bite opening, the sample in this study had an OVB of 5.03 mm (95% CI, 4.80–5.27 mm) at the beginning of the treatment. At the end of the treatment, the OVB was reduced to 3.03 mm (95% CI, 2.66–3.40 mm), with a Prescription aiming to achieve an OVB of 1.80 mm (95% CI, 1.54–2.06 mm). On average about 39% of the initial OVB was reduced in the post‐treatment and only 44% of patients had an OVB < 3 mm after the OVB reduction phase. The average accuracy observed was 61% (95% CI, 52%–69%), which is higher than the literature findings from studies evaluating OVB reduction using clear aligners. In 2021, Blundell et al. found that ClinCheck overpredicted OVB reduction in 95.3% of patients, with an average expression of only 39.2% of the prescribed OVB reduction [23]. This result was recently confirmed by Meade et al., who stated that planned OVB reduction with the Invisalign appliance is challenging for both adults [35] and adolescents. For the latter, less than 60% of the planned OVB changes were achieved [36].

Kravitz et al. [37] found a correlation between patient age and the accuracy of movements, which contrasts with the current result. Specifically, they discovered that Invisalign produced significantly more accurate results for mandibular incisor intrusion in adolescents than in adults, reducing overcorrections for deep overbites in younger patients while applying the mechanics of the reverse curve of Spee. Blundell's study, however, agrees with the result of the present study, showing no difference in the difference of OVB reduction between patients < 14 years and those aged 14–17 years [27]. In this study accuracy of first premolar was the only statistically significant value.

The treatment of the cases in question did not include overcorrections for either the OVB or the COS. As far as staging was concerned, no particular sequence, such as Frog staging, was applied.

However, given the low accuracy, all the authors agree on the need for overcorrections during the treatment planning with ClinCheck, and this aspect could be further investigated in subsequent studies.

Regarding refinements, in this study, all measurements were taken on the post‐treatment digital model at the end of the COS levelling and OVB reduction phase, before any potential refinements. This is because, as highlighted by Kang et al., the majority of overbite correction occurs during the first set of aligners. However additional refinement could improve deep bite correction [38].

This study presents some limitations. Being a retrospective study, its design carries a natural risk of selection bias, which was minimised by excluding subjects who were non‐compliant or non‐attenders. Further limitations are related to the manual landmark identification by the examiner, but the ICC data support the method's reliability. Another area for improvement in the method pertains to the inaccuracy of the OC used as a reference plane to measure the extrusion/intrusion movement of teeth. The method is, nevertheless, validated by the literature [22, 32, 33]; therefore, it is used in this study. Auxiliaries played a significant role in the treatment outcomes. In contrast to other studies that excluded cases involving auxiliaries [23, 26], this study demonstrated that the use of auxiliaries allowed participants to achieve an accuracy level of 61%. Finally, it is important to note that this study exclusively focused on one aligner typology. Further research is needed to compare different protocols and aligner brands, as well as to investigate the amount of overcorrections required [31].

## 5 Conclusions

To summarise the outcomes of this study on the predictability of OVB correction and COS levelling using clear aligners, the following can be concluded:

1. The mean COS levelling accuracy produced by the Invisalign appliance was 62%.
2. The mean OVB reduction accuracy produced by the Invisalign appliance was 61%.
3. Accuracy is not influenced by the age of the patients.
4. Overcorrections are necessary to improve the accuracy of the movements.

## Ethics Statement

The Ethics Committee of the University of Naples Federico II (Italy) approved the research protocols with the following codes: 1792023/1812023.

## Conflicts of Interest

The authors declare no conflicts of interest.

## Data Availability

The data that support the findings of this study are available on request from the corresponding author. The data are not publicly available due to privacy or ethical restrictions.

## References

[1] T. Baccetti , L. Franchi , V. Giuntini , C. Masucci , A. Vangelisti , and E. Defraia , “Early vs. Late Orthodontic Treatment of Deepbite: A Prospective Clinical Trial in Growing Subjects,” American Journal of Orthodontics and Dentofacial Orthopedics 142, no. 1 (2012): 75–82, 10.1016/J.AJODO.2012.02.024. 22748993

[2] M. M. El‐Dawlatly , M. M. S. Fayed , and Y. A. Mostafa , “Deep Overbite Malocclusion: Analysis of the Underlying Components,” American Journal of Orthodontics and Dentofacial Orthopedics 142, no. 4 (2012): 473–480, 10.1016/J.AJODO.2012.04.020. 22999670

[3] J. G. Ghafari , A. T. Macari , and R. V. Haddad , “Deep Bite: Treatment Options and Challenges,” Seminars in Orthodontics 19, no. 4 (2013): 253–266, 10.1053/J.SODO.2013.07.005.

[4] F. Tamburrino , V. D'Antò , R. Bucci , G. Alessandri‐Bonetti , S. Barone , and A. V. Razionale , “Mechanical Properties of Thermoplastic Polymers for Aligner Manufacturing: In Vitro Study,” Dental Journal 8, no. 2 (2020): 47, 10.3390/DJ8020047. PMC7345642 32397575

[5] A. C. Pango Madariaga , R. Bucci , R. Rongo , V. Simeon , V. D'Antò , and R. Valletta , “Impact of Fixed Orthodontic Appliance and Clear Aligners on the Periodontal Health: A Prospective Clinical Study,” Dentistry Journal 8 (2020): 04, 10.3390/DJ8010004. PMC7175220 31906577

[6] V. D'Antò , R. Bucci , V. De Simone , L. H. Ghislanzoni , A. Michelotti , and R. Rongo , “Evaluation of Tooth Movement Accuracy With Aligners: A Prospective Study,” Materials 15, no. 7 (2022): 2646, 10.3390/MA15072646. 35407978 PMC9000684

[7] S. Dianiskova , R. Rongo , R. Buono , L. Franchi , A. Michelotti , and V. D'Antò , “Treatment of Mild Class II Malocclusion in Growing Patients With Clear Aligners Versus Fixed Multibracket Therapy: A Retrospective Study,” Orthodontics & Craniofacial Research 25, no. 1 (2022): 96–102, 10.1111/OCR.12500. 34013659 PMC9290977

[8] R. Rongo , S. Dianišková , A. Spiezia , R. Bucci , A. Michelotti , and V. D'Antò , “Class II Malocclusion in Adult Patients: What Are the Effects of the Intermaxillary Elastics With Clear Aligners? A Retrospective Single Center One‐Group Longitudinal Study,” Journal of Clinical Medicine 11, no. 24 (2022): 7333, 10.3390/JCM11247333. 36555949 PMC9782913

[9] M. Greco and A. Rombolà , “Precision Bite Ramps and Aligners: An Elective Choice for Deep Bite Treatment,” Journal of Orthodontics 49, no. 2 (2022): 213–220, 10.1177/14653125211034180. 34313155

[10] D. Henick , W. Dayan , R. Dunford , S. Warunek , and T. Al‐Jewair , “Effects of Invisalign (G5) With Virtual Bite Ramps for Skeletal Deep Overbite Malocclusion Correction in Adults,” Angle Orthodontist 91, no. 2 (2021): 164–170, 10.2319/072220-646.1. 33434276 PMC8028480

[11] N. D. Kravitz , B. Kusnoto , E. BeGole , A. Obrez , and B. Agran , “How Well Does Invisalign Work? A Prospective Clinical Study Evaluating the Efficacy of Tooth Movement With Invisalign,” American Journal of Orthodontics and Dentofacial Orthopedics 135, no. 1 (2009): 27–35, 10.1016/J.AJODO.2007.05.018. 19121497

[12] V. D'Antò , R. Valletta , R. Ferretti , R. Bucci , R. Kirlis , and R. Rongo , “Predictability of Maxillary Molar Distalization and Derotation With Clear Aligners: A Prospective Study,” International Journal of Environmental Research and Public Health 20, no. 4 (2023): 2941, 10.3390/IJERPH20042941. 36833638 PMC9957205

[13] V. D'Antò , R. Valletta , L. Di Mauro , F. Riccitiello , R. Kirlis , and R. Rongo , “The Predictability of Transverse Changes in Patients Treated With Clear Aligners,” Materials 16, no. 5 (2023): 1910, 10.3390/MA16051910. 36903025 PMC10004392

[14] N. Haouili , N. D. Kravitz , N. R. Vaid , D. J. Ferguson , and L. Makki , “Has Invisalign Improved? A Prospective Follow‐Up Study on the Efficacy of Tooth Movement With Invisalign,” American Journal of Orthodontics and Dentofacial Orthopedics 158, no. 3 (2020): 420–425, 10.1016/J.AJODO.2019.12.015. 32620479

[15] M. P. Muro , A. C. A. Caracciolo , M. P. Patel , M. F. N. Feres , and M. G. Roscoe , “Effectiveness and Predictability of Treatment With Clear Orthodontic Aligners: A Scoping Review,” International Orthodontics 21, no. 2 (2023): 100755, 10.1016/J.ORTHO.2023.100755. 37086643

[16] O. Charalampakis , A. Iliadi , H. Ueno , D. R. Oliver , and K. B. Kim , “Accuracy of Clear Aligners: A Retrospective Study of Patients Who Needed Refinement,” American Journal of Orthodontics and Dentofacial Orthopedics 154, no. 1 (2018): 47–54, 10.1016/J.AJODO.2017.11.028. 29957318

[17] M. Zheng , R. Liu , Z. Ni , and Z. Yu , “Efficiency, Effectiveness and Treatment Stability of Clear Aligners: A Systematic Review and Meta‐Analysis,” Orthodontics & Craniofacial Research 20, no. 3 (2017): 127–133, 10.1111/OCR.12177. 28547915

[18] L. Galan‐Lopez , J. Barcia‐Gonzalez , and E. Plasencia , “A Systematic Review of the Accuracy and Efficiency of Dental Movements With Invisalign,” Korean Journal of Orthodontics 49, no. 3 (2019): 140–149, 10.4041/KJOD.2019.49.3.140. 31149604 PMC6533182

[19] L. Robertson , H. Kaur , N. C. F. Fagundes , D. Romanyk , P. Major , and M. C. Flores , “Effectiveness of Clear Aligner Therapy for Orthodontic Treatment: A Systematic Review,” Orthodontics & Craniofacial Research 23, no. 2 (2020): 133–142, 10.1111/OCR.12353. 31651082

[20] T. Grünheid , C. Loh , and B. E. Larson , “How Accurate Is Invisalign in Nonextraction Cases? Are Predicted Tooth Positions Achieved?,” Angle Orthodontist 87, no. 6 (2017): 809–815, 10.2319/022717-147.1. 28686090 PMC8317555

[21] G. Rossini , S. Parrini , T. Castroflorio , A. Deregibus , and C. L. Debernardi , “Efficacy of Clear Aligners in Controlling Orthodontic Tooth Movement: A Systematic Review,” Angle Orthodontist 85, no. 5 (2015): 881–889, 10.2319/061614-436.1. 25412265 PMC8610387

[22] S. Goh , C. Dreyer , and T. Weir , “The Predictability of the Mandibular Curve of Spee Leveling With the Invisalign Appliance,” American Journal of Orthodontics and Dentofacial Orthopedics 162, no. 2 (2022): 193–200, 10.1016/J.AJODO.2021.04.034. 35753894

[23] H. L. Blundell , T. Weir , B. Kerr , and E. Freer , “Predictability of Overbite Control With the Invisalign Appliance,” American Journal of Orthodontics and Dentofacial Orthopedics 160, no. 5 (2021): 725–731, 10.1016/J.AJODO.2020.06.042. 34373153

[24] N. Shahabuddin , J. Kang , and H. H. Jeon , “Predictability of the Deep Overbite Correction Using Clear Aligners,” American Journal of Orthodontics and Dentofacial Orthopedics 163, no. 6 (2023): 793–801, 10.1016/J.AJODO.2022.07.019. 36681525

[25] H. Burashed and S. R. El , “Quantifying the Efficacy of Overbite Reduction in Patients Treated With Clear Aligners Using Optimized Versus Conventional Attachments,” Journal of the World Federation of Orthodontists 12, no. 3 (2023): 105–111, 10.1016/J.EJWF.2023.03.003. 37142480

[26] H. L. Blundell , T. Weir , and G. Byrne , “Predictability of Overbite Control With the Invisalign Appliance Comparing SmartTrack With Precision Bite Ramps to EX30,” American Journal of Orthodontics and Dentofacial Orthopedics 162, no. 2 (2022): e71–e81, 10.1016/J.AJODO.2022.05.012. 35750579

[27] H. L. Blundell , T. Weir , and M. J. Meade , “Deep Overbite Reduction in Adolescent Patients Treated With Invisalign: A Retrospective Analysis,” American Journal of Orthodontics and Dentofacial Orthopedics 166 (2024): 515–523, 10.1016/J.AJODO.2024.07.008. 39140923

[28] F. Husain , S. Warunek , A. Gurav , T. Giangreco , W. Tanberg , and T. Al‐Jewair , “Influence of Invisalign Precision Bite Ramp Utilization on Deep Bite Correction and Root Length in Adults,” Angle Orthodontist 94, no. 5 (2024): 488–495, 10.2319/012724-70.1. 39230020 PMC11363982

[29] L. Zhang , B. Gong , X. Xie , et al., “The Effectiveness of Clear Aligners in Levelling the Curve of Spee and Related Maxillofacial Factors in Patients With a Deep Overbite,” Australasian Orthodontic Journal 40, no. 1 (2024): 169–177, 10.2478/AOJ-2024-0015.

[30] C. Domenico , F. Carlotta , S. Carmela , et al., “Curve of Spee Modification in Different Vertical Skeletal Patterns After Clear Aligner Therapy: A 3D Set‐Up Retrospective Study,” Progress in Orthodontics 25, no. 1 (2024): 1–8, 10.1186/S40510-023-00503-1/TABLES/7. 38246933 PMC10800316

[31] M. Moshiri , N. D. Kravitz , J. Nicozisis , and S. Miller , “Invisalign Eighth‐Generation Features for Deep‐Bite Correction and Posterior Arch Expansion,” Seminars in Orthodontics 27, no. 3 (2021): 175–178, 10.1053/j.sodo.2021.09.002.

[32] S. M. Adel , N. R. Vaid , N. El‐Harouni , H. Kassem , and A. R. Zaher , “Digital Model Superimpositions: Are Different Software Algorithms Equally Accurate in Quantifying Linear Tooth Movements?,” BMC Oral Health 22, no. 1 (2022): 1–12, 10.1186/S12903-022-02129-X/TABLES/2. 35361187 PMC8973572

[33] S. Braun , W. P. Hnat , and B. E. Johnson , “The Curve of Spee Revisited,” American Journal of Orthodontics and Dentofacial Orthopedics 110, no. 2 (1996): 206–210, 10.1016/S0889-5406(96)70110-5. 8760848

[34] T. Castroflorio , A. Sedran , S. Parrini , et al., “Predictability of Orthodontic Tooth Movement With Aligners: Effect of Treatment Design,” Progress in Orthodontics 24, no. 1 (2023): 2, 10.1186/S40510-022-00453-0. 36642743 PMC9840984

[35] M. J. Meade and T. Weir , “Predicted and Achieved Overjet and Overbite Measurements With the Invisalign Appliance: A Retrospective Study,” Angle Orthodontist 94, no. 1 (2024): 3–9, 10.2319/030923-161.1. 37839803 PMC10928945

[36] M. J. Meade and T. Weir , “Planned and Achieved Overjet and Overbite Changes Following an Initial Series of Invisalign Aligners: A Retrospective Study of Adolescent Patients,” International Orthodontics 22, no. 3 (2024): 100888, 10.1016/J.ORTHO.2024.100888. 38805975

[37] N. D. Kravitz , I. Hansa , N. R. Vaid , M. Moshiri , and S. M. Adel , “Does Age Influence Deep Overbite Correction With Invisalign? A Prospective Study Evaluating Mandibular Incisor Intrusion in Adolescents vs. Adults,” Angle Orthodontist 9 (2023): 145–150, 10.2319/050223-320.1. PMC10893929 37939782

[38] J. Kang , H. H. Jeon , and N. Shahabuddin , “Does Aligner Refinement Have the Same Efficiency in Deep Bite Correction?: A Retrospective Study,” BMC Oral Health 24, no. 1 (2024): 338, 10.1186/s12903-024-04099-8. 38491450 PMC10943900

